# Left Innominate Artery With Anomalous Origin From the Pulmonary Trunk: A Case Report of an Unnoticed Malformation

**DOI:** 10.7759/cureus.79661

**Published:** 2025-02-25

**Authors:** Juan D Ayala Torres, Marcia Mejía Velasquez, Mónica Royero-Arias

**Affiliations:** 1 Radiology, Universidad de Antioquia, Medellín, COL; 2 Radiology, Clínica las Américas, Medellín, COL; 3 Pediatric Radiology, Servicios de Salud San Vicente Fundación, Medellín, COL

**Keywords:** aortic arch, cardiology imaging, cardiovascular abnormalities, computed tomography angiography (cta), diagnostic ct imaging, pediatrics

## Abstract

Thoracic vascular congenital anomalies are malformations with significant clinical implications. One of the rarest anomalies is the anomalous origin of the brachiocephalic trunk from the pulmonary trunk, often associated with serious complications such as pulmonary hypertension. Diagnosis is facilitated by advanced imaging techniques, including MRI and CT scans. We present a case of a premature newborn with congenital heart disease (hypoplastic right ventricle) who developed respiratory and septic complications. A CT angiography revealed a left innominate artery arising from the pulmonary trunk. Despite valvuloplasty and multidisciplinary management, the infant passed away at two and a half months due to multi-organ failure. Early diagnosis and interdisciplinary management, aided by advanced imaging techniques such as CT angiography, are crucial for improving outcomes in patients with complex congenital heart malformations.

## Introduction

Thoracic congenital vascular anomalies encompass a diverse group of malformations affecting blood vessels, which can have significant clinical implications. These anomalies are particularly relevant in the context of congenital heart diseases and genetic syndromes such as "coloboma, heart, atresia of the choanae, retardation of growth and development, genital and urinary anomalies, and ear anomalies" (CHARGE) syndrome, DiGeorge syndrome, and Down syndrome [1,2]. In these conditions, defects in the development of blood vessels and the heart can lead to alterations in blood flow, which, if undiagnosed and untreated, may result in severe complications.

One of the rarest malformations within this group is the anomalous origin of the brachiocephalic trunk from the pulmonary trunk. This is an extremely uncommon defect, with only a few cases reported worldwide. This anomaly is frequently associated with other cardiovascular abnormalities, such as a right aortic arch and cardiac septal defects (malformations affecting the walls separating the heart chambers) [3,4]. Identifying this condition is crucial, as it can lead to significant haemodynamic disturbances, meaning disruptions in the normal dynamics of blood flow within the body. Some of the most severe complications include pulmonary hypertension (abnormally increased pressure in the pulmonary blood vessels) and vascular steal syndrome, in which blood flow is diverted from its normal route, depriving certain tissues of an adequate oxygen supply [3].

To accurately assess these anomalies and determine the best therapeutic approach, advanced medical imaging techniques are employed. Among these, magnetic resonance imaging (MRI) and computed tomography angiography (CTA) are key tools. These techniques provide highly detailed images of the blood vessels and the heart without the need for invasive procedures, thus enabling early diagnosis and effective surgical planning when necessary [5,6]. The use of such technologies is essential for clinical decision-making, particularly in patients with complex anatomical presentations [2].

This case report describes an extremely rare congenital vascular anomaly: an isolated left brachiocephalic trunk. This condition underscores the importance of timely diagnosis, as it allows for appropriate surgical planning. A well-structured treatment approach can prevent severe long-term complications and significantly improve the patient’s quality of life.

## Case presentation

A preterm newborn was diagnosed with congenital heart disease, specifically hypoplastic right ventricle syndrome, via prenatal ultrasound, leading to delivery by caesarean section at 34 weeks of gestation. The APGAR scores were 7 at one minute, 8 at five minutes, and 9 at ten minutes. Birth weight was 2,445 gm, with a length of 43 cm. The neonate developed neonatal respiratory distress syndrome, congenital pneumonia, and early-onset sepsis, as evidenced by radiological findings, elevated inflammatory markers, and a critical condition requiring invasive mechanical ventilation, circulatory support, and antibiotic therapy.

**Figure 1:**
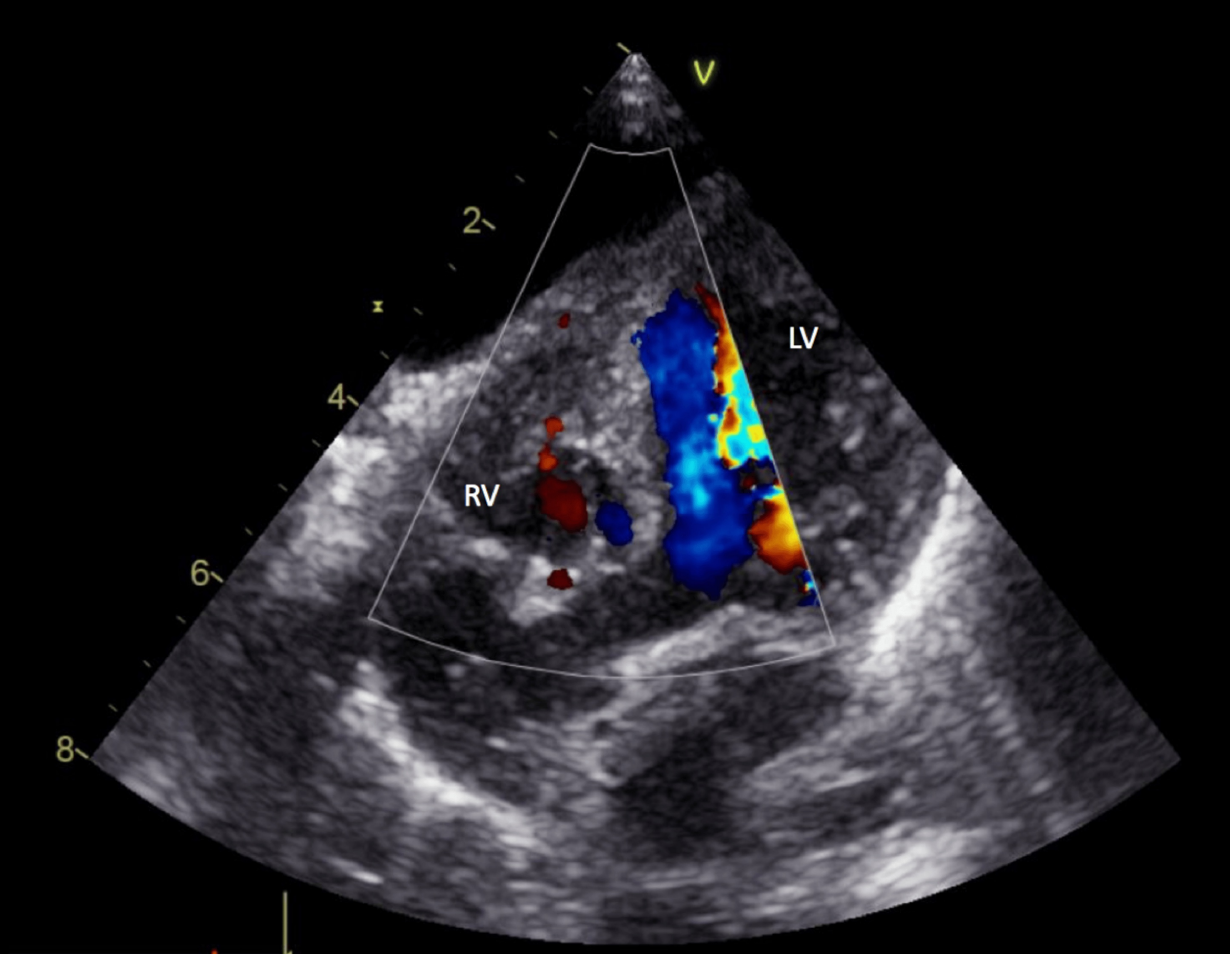
Echocardiography. Four-chamber echocardiographic view showing a hypoplastic right ventricle (RV: right ventricle, LV: left ventricle).

At one week of life, the infant was transferred to a higher-complexity hospital with paediatric cardiology and cardiovascular surgery facilities. An echocardiogram confirmed a hypoplastic right ventricle, tricuspid valve hypoplasia, severe valvular pulmonary stenosis, and a patent ductus arteriosus (PDA) with no restriction, along with a patent foramen ovale (PFO) without obstruction and a right aortic arch (Figure 1).

Initially, the patient received non-invasive ventilatory support and alprostadil (0.02 mcg/kg/min) to maintain ductal patency. At 29 days of life, after reaching the appropriate weight for palliative surgery, a Blalock-Taussig (BT) shunt was planned. However, during the procedure, a complex supra-aortic vascular anatomy was encountered, preventing the completion of the surgery. Consequently, a CT angiography was requested to clarify the intraoperative findings.

**Figure 2:**
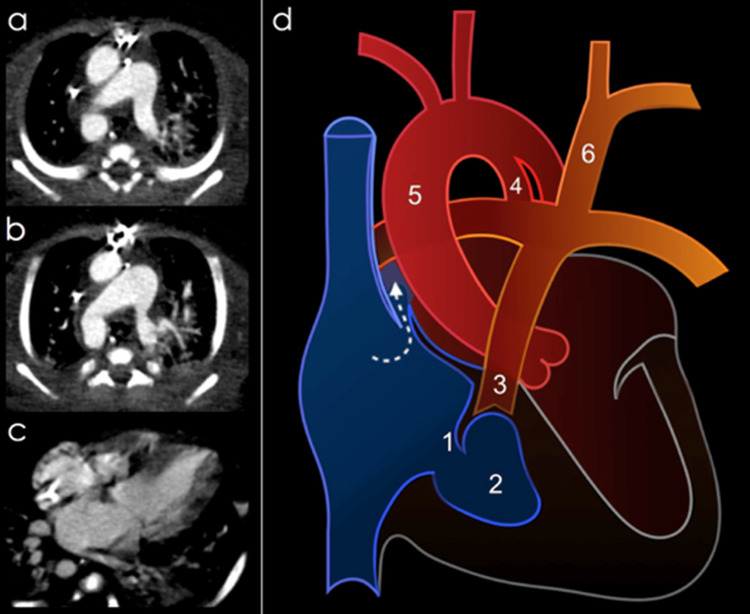
Axial slices of thoracic CT angiography presented in descending order. a) right aortic arch, b) patent ductus arteriosus, c) hypoplastic right ventricle, d) diagram of the identified malformations (made by authors, arrow indicating patent foramen ovale, 1: hypoplastic tricuspid valve, 2: hypoplastic right ventricle, 3: pulmonary stenosis, 4: patent ductus arteriosus, 5: right aortic arch, 6: left innominate artery emerging from the main trunk of the pulmonary artery).

**Figure 3:**
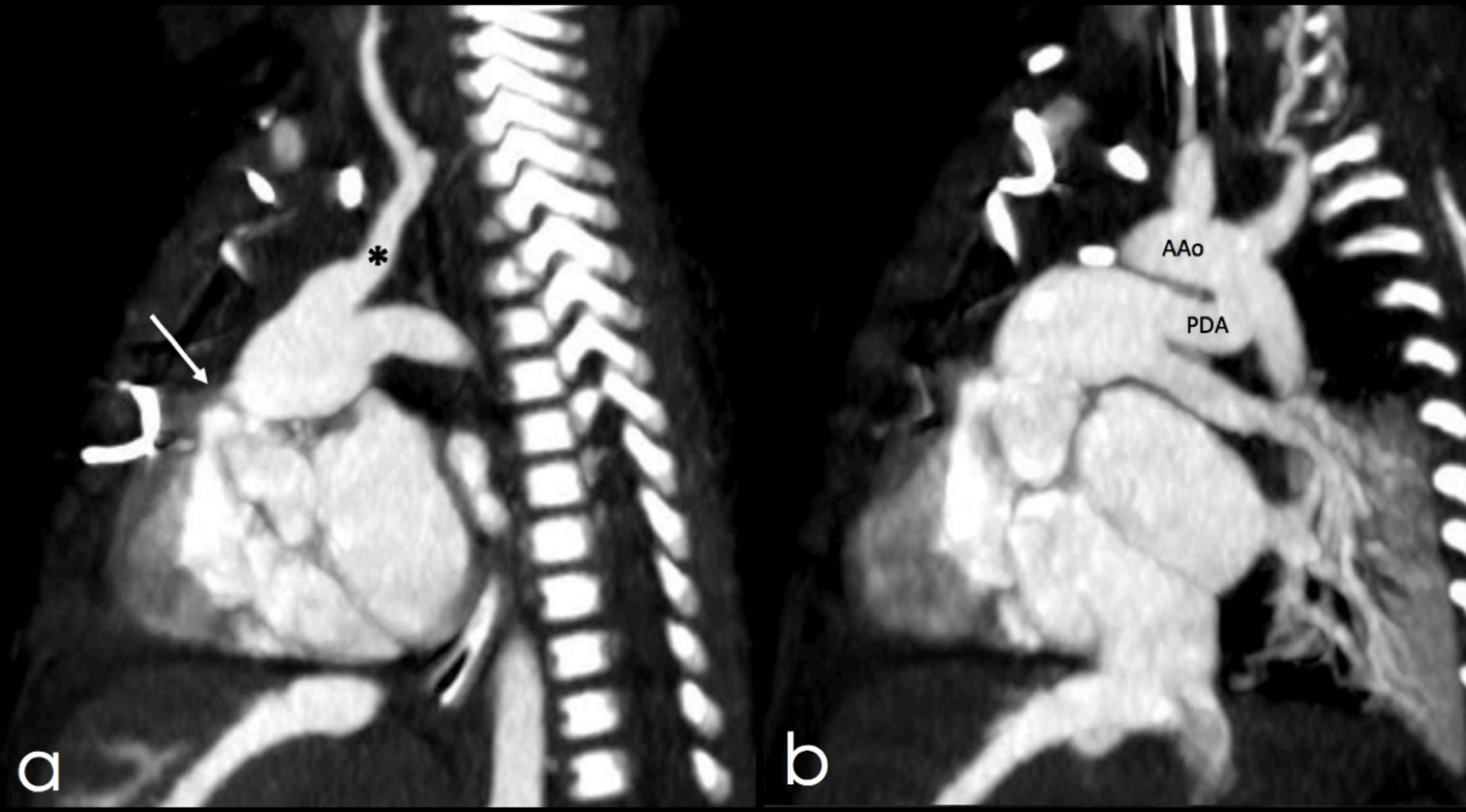
Sagittal slices of thoracic CT angiography presented from left to right. a) pulmonary valve stenosis (white arrow) and left innominate artery (*) originating from the pulmonary trunk, b) patent ductus arteriosus (PDA) and aortic arch (AAo).

The CT angiography revealed a previously undetected left innominate artery arising from the pulmonary trunk via a ductus, an incidental finding overlooked in previous studies (Figures 2, 3).

Subsequently, the patient experienced haemodynamic decompensation with a right ventricular infarction, exacerbating her heart failure. She developed severe oedematous syndrome with bilateral pleural effusions, ascites, and pericardial effusion, necessitating evacuative thoracentesis. Additionally, she was complicated by an infection caused by extended-spectrum beta-lactamase (ESBL)-producing Klebsiella pneumoniae, isolated in pleural fluid and tracheal aspirate.

**Figure 4:**
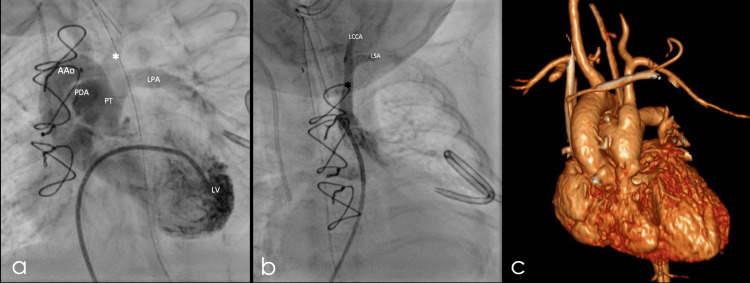
Left innominate artery. a) and b) coronal slices of cardiac catheterization confirming the CT findings of the left innominate artery (*), c) 3D reconstruction of CT angiography in the frontal plane demonstrating the same findings. LV: left ventricle, AAo: aortic arch, PDA: patent ductus arteriosus, PT: pulmonary trunk, LPA: left pulmonary artery, LCCA: left common carotid artery, LSA: left subclavian artery.

At two months of age, she underwent a cardiac catheterization, which confirmed the tomographic findings. A pulmonary valvuloplasty was performed, successfully reducing pulmonary pressure to 50% of the systemic level (Figure 4).

Despite these measures, the patient remained in a critical condition, requiring ventilatory support, prostaglandin infusion, and vasopressor agents. Multidisciplinary management involved specialists in paediatric cardiology, cardiovascular surgery, intensive care, and nephrology. Ultimately, at two and a half months of age, the patient developed multiorgan dysfunction with refractory septic and cardiogenic shock, was unresponsive to catecholamines, and passed away following the terminal progression of her cardiac disease.

## Discussion

Isolated left brachiocephalic trunk is an extremely rare condition, with very few cases reported in the medical literature [1,3]. This type of malformation is commonly associated with other congenital anomalies of the aortic arch and intracardiac defects [2,3,6-9]. In many reported cases, the anomaly appears in combination with right aortic arches, and persistent ductus arteriosus, and, in some instances, is accompanied by CHARGE syndrome or other anomalies such as septal defects or aberrant arteries [1-3,6,9]. The literature highlights the importance of advanced imaging for diagnosis, with echocardiography, computed tomography angiography (CTA), and magnetic resonance imaging (MRI) being essential tools [2,10]. Management of this condition generally includes surgical intervention to correct circulation and prevent haemodynamic complications, especially when a patent ductus arteriosus (PDA) or associated stenosis is present [2,3].

The origin of this malformation stems from disruptions in the embryonic development of the aortic arches. Edwards' model (double aortic arch) may explain the anomaly’s origin, suggesting that interruption of certain segments during embryonic development can lead to various malformations [7,8]. During the early weeks of gestation, the embryonic arterial system forms from six pairs of aortic arches that connect the primitive ventral and dorsal aortas. The brachiocephalic trunk normally develops from the fourth aortic arch, with the left arch contributing to the formation of the main aortic arch and the right arch forming part of the subclavian artery [1,3]. If this process of regression and development does not occur correctly, multiple vascular system malformations may arise [7].

Specifically, this malformation is characterized by the disconnection of the left brachiocephalic trunk from the ascending aorta, with a possible anomalous connection to the pulmonary trunk, usually through a patent ductus arteriosus [2]. The causes include defects in the migration of neural crest cells, which are essential for the proper formation of the great vessels [1,10]. Errors in this migration can result in incomplete formation of the aortic arches and the emergence of these anatomical variants [2]. Additionally, this anomaly is often associated with other congenital conditions, such as right aortic arch and septal defects, suggesting a possible genetic basis in some cases [2,3,11].

Cases reported in the literature include patients with congenital vascular anomalies or complex congenital heart disease, presenting with symptoms ranging from severe hypoxaemia and vascular congestion to more subtle manifestations such as failure to thrive and respiratory symptoms [4]. Associated anomalies described include septal and valvular defects, with isolated vascular malformations without accompanying cardiac anomalies being rare [8]. Although most cases in the literature are diagnosed later in life, it has been demonstrated that early management as the identification and treatment of the anomaly within the first few months of life, ideally during the neonatal period significantly improves prognosis and prevents long-term complications [2,3]. A notable example is a patient with an anomalous origin of the left brachiocephalic trunk from the pulmonary trunk, in whom surgical intervention performed on day 30 of life resulted in successful correction, restored perfusion, and discharge in a satisfactory condition [3]. Similarly, early reimplantation of the left brachiocephalic trunk into the aorta has proven to be an effective treatment, restoring normal blood flow and eliminating left-to-right shunting, with a favourable long-term prognosis [2]. These cases highlight the importance of early intervention in congenital vascular anomalies to optimize clinical outcomes.

Echocardiography remains the primary tool for diagnosing and assessing congenital cardiac malformations; however, it has limitations in visualizing the supra-aortic vascular structures in detail. Advanced imaging techniques, such as CTA, MRI, and arteriography, are essential for accurately identifying these malformations and planning surgical interventions. These tools provide a detailed visualization of the vascular anatomy, which is crucial for guiding therapeutic decisions [2,3]. In the case of this patient, such imaging studies were instrumental in understanding the severity of the condition, ultimately contributing to the unfavourable outcome.

## Conclusions

Interdisciplinary management and early detection through imaging diagnostics are essential for surgical planning and improving outcomes in patients with complex congenital heart malformations. Early detection involves identifying structural anomalies from the neonatal period to early childhood, allowing for timely interventions that help prevent long-term complications. Non-invasive imaging techniques such as computed tomography angiography (CTA) and magnetic resonance imaging play a crucial role in assessing vascular anomalies with high precision, facilitating safer and more effective surgical planning. CTA, in particular, provides clear anatomical visualization, enhancing decision-making and minimizing intraoperative risks. Integrating these advanced diagnostic tools into early management strategies improves surgical safety, optimizes postoperative outcomes, and reduces morbidity, ultimately contributing to a better quality of life for affected patients.
